# Exploring the Therapeutic Potential of N-Methyl-D-Aspartate Receptor Antagonists in Neuropathic Pain Management

**DOI:** 10.3390/ijms252011111

**Published:** 2024-10-16

**Authors:** Ciprian Pușcașu, Cornel Chiriță, Simona Negreș, Nicoleta Mirela Blebea

**Affiliations:** 1 Faculty of Pharmacy, “Carol Davila” University of Medicine and Pharmacy, Traian Vuia 6, 020956 Bucharest, Romania; ciprian.puscasu@umfcd.ro (C.P.); simona.negres@umfcd.ro (S.N.); 2 Faculty of Pharmacy, “Ovidius” University of Constanța, Căpitan Aviator Al. Şerbănescu 6, 900470 Constanța, Romania; nicoleta.blebea@gmail.com

**Keywords:** neuropathic pain, NMDA receptor antagonist, ketamine, memantine, methadone

## Abstract

Neuropathic pain (NeP) is a complex and debilitating condition that impacts millions of people globally. Although various treatment options exist, their effectiveness is often limited, and they can be accompanied by significant side effects. In recent years, there has been increasing interest in targeting the N-methyl-D-aspartate receptor (NMDAR) as a potential therapeutic approach to alleviate different types of neuropathic pain. This narrative review aims to provide a comprehensive examination of NMDAR antagonists, specifically ketamine, memantine, methadone, amantadine, carbamazepine, valproic acid, phenytoin, dextromethorphan, riluzole, and levorphanol, in the management of NeP. By analyzing and summarizing current preclinical and clinical studies, this review seeks to evaluate the efficacy of these pharmacologic agents in providing adequate relief for NeP.

## 1. Introduction

Neuropathic pain (NeP) is “pain arising as a direct consequence of a lesion or disease of the somatosensory system” and is characterized by a paradoxical association with sensory loss [1]. NeP can be caused by traumatic nerve, spinal cord, or brain injury (including stroke) or may be linked to various conditions like diabetes, infection, multiple sclerosis [2], or cancer, as well as the harmful impact of chemotherapy drugs [3,4].

A systematic review of prevalence studies based on the general population determined that the prevalence of pain with neuropathic characteristics falls within the range of 7% to 10% [5]. Furthermore, recent research indicated that the age-standardized prevalence of chronic polyneuropathy is 3.3% for the European Union, 3.0% for the United States, and 2.3% for the global population and is projected to rise by approximately 25% over the next 20 years, taking into account anticipated age distribution [6].

Effective treatment of NeP presents significant challenges and is associated with significant decreases in quality of life, along with a substantial economic burden [7]. Patients suffering from chronic NeP typically experience poorer physical and mental health in comparison to those with other forms of chronic pain, even after adjusting the pain intensity [8,9,10]. The link to diminished physical and mental health implies that the quality of life is negatively impacted by the nature rather than just the intensity of NeP, highlighting the need for a comprehensive treatment approach [11]. Managing NeP can be challenging and often involves a trial-and-error process [12]. Conventional analgesics may not effectively relieve pain for NeP patients [13,14]. In a survey, NeP patients were more likely to use opioids and multiple pain medications but reported less pain relief [15]. However, it has been shown that adjunctive therapy with cannabidiol may have therapeutic potential in neuropathic pain [16]. First-line therapies for NeP, like tricyclic antidepressants, selective inhibitors of serotonin and norepinephrine reuptake, and gabapentinoids, require careful dosing due to potential side effects, particularly in elderly patients [17,18].

Nevertheless, in recent years there has been growing interest in antagonists targeting the presynaptic N-methyl-D-aspartate receptor (NMDAR) to alleviate pain from various types of NeP. The NMDAR is the most complex glutamatergic receptor, and its hyper/hypofunction leads to the development of various neurological disorders [19]. It is a ligand-dependent receptor, widely distributed in the brain and spinal cord, especially in the hippocampus and cerebral cortex, whose activation depends on the levels of glutamate and glycine. However, this ionic channel is the only one that allows the conduction of calcium (Ca^2+^), sodium (Na^+^), and potassium (K^+^) [20]. Research indicates that the NMDAR within the dorsal horn is significant in both inflammation and central sensitization induced by nerve injury [21]. Activation of the NMDAR leads to disruptions in the sensory system, both peripheral and central, causing neuronal excitation and abnormal pain symptoms such as spontaneous pain, allodynia, and hyperalgesia [22,23]. While central NMDARs, particularly those in the spinal cord, remain a focus of study, growing evidence indicates that NMDARs in peripheral tissues and visceral pain pathways also contribute significantly to nociception. This suggests that, in chronic pain conditions, NMDAR activation occurs at various levels of the neural axis, making each level a potential target for therapeutic intervention [23].

Primary afferent neurons in the spinal dorsal horn release various neurotransmitters, including glutamate, IL-1β (interleukin 1β), ATP (adenosine triphosphate), TNF-α (tumor necrosis factor α), and NGF (nerve growth factor). Glutamate and IL-1β activate NMDAR on secondary neurons, causing Ca^2+^ influx and activating AMPARs (α-amino-3-hydroxy-5-methyl-4-isoxazolepropionic acid receptors), which reduces GABA (gamma-aminobutyric acid) release from inhibitory interneurons. ATP activates the P2Y4 receptor on secondary neurons, further stimulating NMDAR and leading to the activation of the JNK (c-Jun N-terminal kinase), P38, and MAPK (mitogen-activated protein kinase) pathways. These processes contribute to central sensitization and synaptic remodeling [24]. NeP resulting from nerve injury involves increased glutamate release from primary afferent terminals, activating AMPA and mGluR5 (metabotropic glutamate receptor 5) receptors in the spinal cord [25,26,27]. Glutamate interacts with postsynaptic receptors for excitatory neurotransmission, while NMDARs can enhance the release of neurotransmitters like substance P in the spinal dorsal horn [28]. Presynaptic NMDARs may boost glutamate release, and μ-opioid receptor stimulation initiates long-term potentiation in pain pathways [29]. Heightened activation of glutamate receptors contributes to the amplification of excitatory synaptic signaling in chronic pain’s nociceptive pathways. Excessive activation of glutamate receptors in spinal dorsal horn neurons is primarily caused by increased glutamate release from primary afferent terminals within the spinal dorsal horn, enhanced numbers and functionality of glutamate receptors, and impaired clearance of glutamate [30,31,32,33]. In normal conditions, the NMDAR is blocked by Mg^2+^ and is only activated briefly [34]. However, in abnormal situations, the removal of Mg^2+^ leads to overactivation of the receptor, causing excessive Ca^2+^ influx and potentially triggering cell death processes like apoptosis or necrosis [35]. Moreover, in NeP, NMDARs are involved in a process called “wind-up”, where repeated stimulation of pain signals leads to an increased response to pain. This phenomenon can result in chronic pain conditions where even non-painful stimuli are perceived as painful [36,37].

Considering these, the main objective of this narrative review is to comprehensively examine the use of the following NMDAR antagonists in managing NeP: ketamine, memantine, methadone, amantadine, carbamazepine, valproic acid, phenytoin, dextromethorphan, riluzole, and levorphanol (Table 1). This review aims to analyze and summarize the current literature, including preclinical and clinical studies, and to evaluate whether specific pharmacologic agents provide adequate relief for NeP, as there is no such review to date.

## 2. Scope

This narrative review analyzes a combined total of 50 preclinical and 52 clinical studies. The findings from these studies are condensed and presented in the following tables in Section 3, arranged in chronological order. Among the NMDAR antagonists investigated in this study, ketamine emerges as the most studied for treating NeP, with 24 studies dedicated to its examination. Conversely, evidence for the efficacy of amantadine and levorphanol is notably scarce.

It should be noted that, in the preclinical studies, the most commonly assessed parameters were mechanical hypersensitivity and hypersensitivity to a hot stimulus. A variety of animal models were used to evaluate the benefits of NMDAR antagonists, including chronic constriction injury (CCI), streptozotocin (STZ)-induced diabetic neuropathy (DN), chemotherapy-induced polyneuropathy (CIPN), spinal cord injury (SCI), and spinal nerve ligation (SNL).

In clinical studies, pain severity was the primary outcome investigated in many of the studies, with the Numeric Rating Scale (NRS) and the Visual Analog Scale (VAS) being the most commonly used pain scales. However, other parameters were also investigated, such as the frequency of pain attacks, paresthesia, allodynia, walking ability, quality of life, depression, and anxiety. The results from clinical studies are based on various types of NeP conditions, including DN, post-herpetic neuralgia (PHN), trigeminal neuralgia (TN), cancer-related neuropathic pain (CRNP), radiculopathy, CIPN, and chronic idiopathic axonal polyneuropathy (CIAP).

## 3. Discussions

Since the late 1980s, it has been recognized that NMDAR antagonists can reduce neuronal hyperexcitability and alleviate pain. Various NMDAR antagonists have been studied in both preclinical and clinical pain research [38]. Despite numerous studies, there remains no consensus on the effectiveness of NMDAR antagonists for NeP, prompting the need for the current narrative review. This section explores the benefits of NMDAR antagonists for NeP treatment based on the analysis of preclinical and clinical studies included in this review.

### 3.1. Ketamine

Ketamine binds non-competitively to NMDARs at the phencyclidine site, affecting receptors through allosteric mechanisms [39]. It equally binds NMDARs subtypes 2A to 2D and surpasses the normal capacity of NMDAR magnesium-dependent gating [40]. Ketamine is rapidly absorbed, with high bioavailability (around 93%), but only 17% is absorbed after first-pass metabolism. It undergoes hepatic metabolism, with norketamine as a major metabolite [39]. Most of the administered dose is recovered in urine as metabolites. Ketamine can also be eliminated through bile and feces [41]. Side effects include apnea, sedation, increased salivary secretions, hallucinations, dizziness, and drowsiness [39,42].

Ketamine, as a potent NMDAR antagonist, effectively blocks the receptor and reduces the neuronal hyperexcitability associated with NeP [43]. Moreover, research shows that ketamine levels in the brain are linked to pain relief in ischemic pain [44]. Additionally, studies suggest that ketamine decreases connectivity in brain regions involved in pain perception and emotional processing [45]. Ketamine is likely the most extensively studied NMDAR antagonist for treating NeP, resulting in the inclusion of the highest number of studies among all NMDAR antagonists in our review. We analyzed over 24 studies that examined the use of ketamine in treating NeP, including 13 preclinical studies and 11 clinical studies (Table 2).

Of the preclinical studies analyzed, 10 out of 13 indicated that ketamine has a positive effect in reducing pain sensitivity associated with NeP. In contrast, Salvat et al. showed that, in a CCI model of neuropathy in mice, ketamine at a dose of 15 mg/kg was effective as a treatment solely during the early postsurgical period [46]. In addition, the studies conducted by Kroin et al. [47] and Humo et al. [48] indicated that the effect of ketamine on NeP is not long-lasting. On the other hand, two studies showcased the anti-inflammatory potential of ketamine by effectively reducing the levels of pro-inflammatory cytokines like TNF-α, IL-6 (interleukin 6), and IL-1β [49,50]. Furthermore, Tai et al. [49] emphasized that enhancing the cage setup with additional space and varied objects for physical activity improved the effectiveness of ketamine in reducing pain sensitivity and promoted tissue integrity and locomotion by targeting the glutamatergic system.

Clinical studies support the results of preclinical research, with 8 out of 11 studies indicating the benefits of ketamine in reducing NeP. Over half of the studies (six studies) utilized intravenous (i.v.) administration, yielding contrasting results. Therefore, three studies documented that ketamine effectively relieved pain [51,52,53], whereas one study indicated that only half of the patients experienced pain reduction 1 month after treatment [54]. On the other hand, two studies reported no notable pain reduction after i.v. treatment with ketamine [55,56]. When given orally, ketamine showed clinically effective outcomes in two studies [57,58]. However, a study conducted by Fallon et al., which was the largest study involving 214 patients with CIPN, demonstrated poor results [59]. In other studies, topical administration [60] and subcutaneous (s.c.) infusion [61] of ketamine were also examined, showing positive results. Crucially, the effects of ketamine varied across different types of NeP, ranging from SCI to dentoalveolar NeP and CRNP. Rabi et al. [60] examined the impact of 10% topical ketamine on five patients with NeP due to SCI over a 2-week treatment period. They found that all patients experienced a decrease in their pain levels, as shown in the NPS. Moreno-Hay et al. [53] reported a case involving a patient with dentoalveolar NeP who underwent five regimens of ketamine infusion over 5 years. The patient’s NeP symptoms were effectively managed, leading to the discontinuation of prior methadone treatment. Provido-Aljibe et al. [61] investigated 41 patients with CRNP, highlighting the positive effects of ketamine on alleviating neuropathic pain. Ketamine was administered at doses ranging from 75 to 475 mg via s.c. infusion, and the pain intensity was assessed using the NPS (Numerical Pain Score). Finally, Martin et al. [58] examined the advantages of incorporating either memantine or dextromethorphan into the treatment regimen of i.v. ketamine for 60 patients with refractory NeP. Dextromethorphan, but not memantine, was observed to extend the pain relief provided by ketamine by up to 1 month, as indicated by VAS and Neuropathic Pain Symptom Inventory (NPSI) ratings.

**Table 2:** Preclinical and clinical studies that evaluated the effect of ketamine on NeP.

Ketamine					
Preclinical Studies					
First Author/Reference	Animals	Animal Model	Dosage	Route/Frequency	Results
M’Dahoma et al. (2015) [62]	Male Sprague–Dawley rats	CCI	50 mg/kg bw	i.p. single dose	Alleviated mechanical hypersensitivity in von Frey test.
Mak et al. (2015) [63]	Male Wistar rats	STZ-induced DN	20 mg/kg bw	s.c. 5-day infusion	Demonstrated antinociceptive action in radiant heat plantar test and tail-flick test that lasted for 4 weeks.
Claudino et al. (2018) [64]	Male Wistar rats	CION	0.5–1 mg/kg bw	intranasal single dose	0.5 mg/kg effectively reversed heat-induced hypersensitivity in the radiant heat test, while 1 mg/kg was found to alleviate mechanical hypersensitivity in the von Frey test.
Doncheva et al. (2018) [65]	Male Wistar rats	CCI	50 mg/kg bw	i.p. single dose	Alleviated hypersensitivity in both hot-plate test and analgesia-meter test.
Pan et al. (2018) [66]	Male Sprague–Dawley rats	SNI	10 mg/kg bw	i.p. single dose	Reversed mechanical hypersensitivity in the von Frey test.
Salvat et al. (2018) [46]	Male 6J mice	CCI	15 mg/kg bw	i.p. 10 days	Provided analgesic effects only in the initial stages after surgery in the von Frey test.
Fang et al. (2019) [67]	Male Sprague–Dawley rats	SNI	10 mg/kg bw	i.p. single dose	Successfully alleviated the mechanical sensitivity in the von Frey test.
Kroin et al. (2019) [47]	Female D1 mice	SNI	10 mg/kg Bw	i.p. single dose	Did not produce long-lasting analgesia in von Frey test.
Humo et al. (2020) [48]	Male C75BL/6 mice	CCI	15 mg/kg bw	i.p. single dose	Provided temporary relief from increased sensitivity to mechanical stimuli in the von Frey test, with effects lasting less than 24 h.
Tai et al. (2021) [49]	Male Sprague–Dawley rats	SCI	30 mg/kg bw	i.m. for 10 days, starting from day 8 after SCI	In combination with environmental enrichment, improved the alleviation of pain in both von Frey test and plantar test, supported tissue health and mobility; reduced activation of MAPK family, NF-κB, and IL-1β signaling, while the levels of excitatory amino acid transporter 2 were restored.
Kim et al. (2022) [68]	Male Wistar rats	PSNL	5–10 mg/kg bw	i.p. 5 weeks, with 2 weeks pause after the first 4 weeks	The higher dose resulted in a significant increase in the mechanical withdrawal threshold during the von Frey test, which lasted for over 2 weeks.
Seo et al. (2023) [69]	Male Sprague–Dawley rats	SNI	50 mg/kg bw	i.p. in the 15, 18, 21 day after SNI	Improved symptoms of NeP in the von Frey test and dry ice test, suppressed the presence of NMDA receptors and ATF-6 expression during ER stress.
Han et al. (2023) [50]	Male Sprague–Dawley rats	CCI	5–15 mg/kg bw	i.p. 14 days	Efficiently alleviated mechanical and thermal hyperalgesia in von Frey and radiant heat tests; decreased TNF-α, IL-6, IL-1β levels and p62 expression; upregulated C3II/LC3I and Beclin1 expression.
**Clinical Studies**					
**First Author/Reference**	**Population**	**Type of NeP**	**Dosage**	**Route/Frequency**	**Results**
Kim et al. (2015) [51]	N = 30	Severe NeP	Ketamine 1 mg/kg bw or Magnesium sulfate 30 mg/kg bw	i.v. for 1 h	Out of 15 patients, 10 recorded pain reduction according to VAS score.
Rabi et al. (2016) [60]	N = 5	SCI patients with NeP	10% cream	topical ×3 times/day 2 weeks	After the 2-week period, all five participants experienced a reduction in their pain levels as indicated in the NPS.
Rigo et al. (2017) [57]	N = 42	Refractory chronic NeP	Ketamine 30 mg or Methadone 3 mg or Methadone 3 mg + Ketamine 30 mg	orally 90 days	Only the group treated with ketamine alone demonstrated a noticeable pain reduction according to VAS and also an alleviation of allodynia.
Fallon et al. (2018) [59]	N = 214	CIPN	40–400 mg	orally 16 days	Showed no significant difference in pain reduction according to Sensory Component of the Short Form McGill Pain Questionnaire.
Czarnetzki et al. (2018) [55]	N = 160	NeP after back surgery	0.25 mg/kg bw preoperatively, 0.25 mg/kg bw intraoperatively, 0.1 mg/kg bw from 1 h before the end of surgery and continuing until the patient’s discharge from the recovery room.	i.v.	The low-dose infusion administered during the perioperative period did not show any impact on the occurrence of neuropathic lower back pain 6 or 12 months after surgery, according to the DN4 questionnaire.
Bosma et al. (2018) [54]	N = 30	Refractory NeP	0.5–2 mg/kg bw	i.v. 6 h/day 5 days	After 1 month post-treatment, about 50% of patients experienced pain reduction according to Brief Pain Inventory questionnaire.
Weber et al. (2018) [52]	N = 1	Bilateral neuropathic leg pain	7 µg/kg/min	i.v. 5 days	Demonstrated fast-acting pain-relieving effects, with 70% reduction of pain, according to rating scale of burning quality, that persisted for a duration of 5 months after the initial administration.
Moreno-Hay et al. (2018) [53]	N = 1	Dentoalveolar NeP	20–50 mg	i.v. 5 infusions over 4 years	The patient’s NeP symptoms were efficiently treated, and methadone was eventually stopped.
Martin et al. (2019) [58]	N = 60	Refractory NeP	Ketamine 0.4–0.5 mg/kg bw, FOLLOWED BY Dextromethorphan 90 mg or Memantine 20 mg	Ketamine i.v. (infusion, 2 h) Dextromethorphan orally, 12 weeks Memantine orally, 12 weeks	Dextromethorphan, not memantine, was found to prolong the pain-relieving effects of ketamine for up to 1 month, according to VAS and NPSI.
Pickering et al. (2020) [56]	N = 20	Refractory chronic NeP	Ketamine 0.5 mg/kg bw or Ketamine 0.5 mg/kg bw + Magnesium sulfate 3 g	One infusion every 35 days for 3 times	At 35 days after infusion, ketamine did not provide pain relief according to four-item Neuropathic Pain Questionnaire; when combined with magnesium, the analgesic effects were not further enhanced.
Provido-Aljibe et al. (2022) [61]	N = 41	CRNP	75–475 mg	s.c. 5 days	Efficiently decreased the pain levels, according to NPS.

ATF-6—activating transcription factor-6; bw—body weight; CCI—chronic constriction injury; CION—constriction of intraorbital nerve; CIPN—chemotherapy-induced polyneuropathy; CRNP—cancer-related neuropathic pain; DN—diabetic neuropathy; DN4—Douleur Neuropathique en 4 Questions; ER—endoplasmic reticulum; i.p.—intraperitoneally; IL-1β—interleukin 1β; IL-6—interleukin 6; i.m.—intramuscular; i.v.—intravenous; MAPK—mitogen activated protein kinase; NF-κB—nuclear factor κB; NMDA—N-methyl-D-aspartate; NPS—numerical pain score; NPSI—Neuropathic Pain Symptom Inventory;; PSNL—partial sciatic nerve ligation; s.c.—subcutaneous; SCI—spinal cord injury; SNI—spared nerve injury; STZ—streptozotocin; TNF-α—tumor necrosis factor α; VAS—Visual Analog Scale.

### 3.2. Dextromethorphan

Dextromethorphan acts as a low-affinity uncompetitive NMDAR antagonist, a sigma-1 receptor agonist, and an antagonist of α3/β4 nicotinic receptors [70,71,72]. It has also been shown to decrease K^+^-stimulated glutamate release, possibly through sigma receptor-related mechanisms [73,74]. Additional functions of dextromethorphan appear to involve mild inhibition of serotonin reuptake through suggested high-affinity binding to the serotonin transporter [75]. The bioavailability of dextromethorphan is both poor and inconsistent. This is due to its rapid first-pass metabolism and subsequent elimination [76]. Dextromethorphan is mainly metabolized into dextrorphan and has a half-life ranging from 3 to 30 h, and potential side effects may include confusion, agitation, memory loss, hallucinations, dysarthria, and ataxia [72,76,77,78].

The initial discovery of dextromethorphan’s neuroprotective properties was made by Choi et al., who showed that the drug reduced glutamate-induced neurotoxicity in neocortical cell cultures [79]. The predominant mechanism underlying the neuroprotective potency is believed to be antagonism of the NMDA receptor/channel complex [80]. Moreover, in in vitro studies, both dextromethorphan and its primary metabolite, dextrorphan, have been shown to block the NMDAR in the central nervous system (CNS) and spinal regions. This action leads to the suppression of NMDA-induced convulsions and the reduction of hypoglycemic neuronal damage, with dextrorphan exhibiting a greater affinity for the NMDAR than dextromethorphan [81,82,83]. Among the four preclinical studies analyzed in this review (Table 3), the findings suggest a favorable outlook for the utilization of dextromethorphan in NeP. Thus, Zbârcea et al. [84] and Fahmi et al. [85] demonstrated the efficacy of dextromethorphan in reversing tactile allodynia when administered orally at a dose of 20 mg/kg and thermal hyperalgesia when administered intrathecally at a dose of 10 nmol. On the other hand, the research findings by Yang et al. [86] emphasized that the antiallodynic effect of dextromethorphan was enhanced when administered alongside oxycodone in mice with SNL. In addition, Shi et al. [87] evaluated the potency of dextromethorphan administered alone and in combination with gapabentin in two different models of NeP (photochemically-induced ischemic SCI and SNI (spared nerve injury)). The results indicated that dextromethorphan alone did not provide any pain relief. In contrast, the combination of dextromethorphan and gabapentin led to complete alleviation of allodynia.

The findings from clinical trials are limited, as only 1 study has explored the antihyperalgesic effects of dextromethorphan in humans (Table 2). Martin et al. [88] investigated the action of the NMDAR antagonist in a freeze-injury-induced hyperalgesia model in healthy volunteers and demonstrated that it reversed sensitization in both peripheral and central neurons.

### 3.3. Memantine

Memantine functions as an NMDAR antagonist with low to moderate affinity, pronounced voltage dependency, and fast blocking and unblocking kinetics [89,90,91]. Additionally, it also acts as an antagonist at the serotonergic type 3 receptors and nicotinic acetylcholine receptors [45]. Memantine is effectively absorbed after oral administration, reaching peak concentrations in 3–8 h [90]. It undergoes partial metabolism in the liver, with the hepatic CYP450 enzyme system playing a minor role. The primary route of excretion is through urine, with around 48% of the administered dose being excreted unchanged [92]. Agitation, constipation, urinary tract infections, diarrhea, headache, and confusion are the main side effects [93,94].

Animal studies indicate that memantine may serve as a promising alternative for treating NeP, as all five reviewed studies demonstrated positive outcomes (Table 4). A study conducted by Ciotu et al. investigated the effect of memantine in an animal model of paclitaxel-induced NeP and showed that the NMDAR antagonist effectively reversed mechanical sensitivity in a dose-dependent manner [95]. Alomar et al. [96] administered a 10 mg/kg dose of memantine to mice with DN, showing that the drug not only effectively reduced thermal and mechanical hypersensitivity but also had the potential to decrease the release of proinflammatory cytokines TNF-α and IL-1β in the spinal cord. The findings of this study are consistent with those of the study carried out by Solmaz et al., which demonstrated the neuroprotective and antioxidant effects of memantine by significantly reducing TNF-α and MDA (malondialdehyde) levels in an animal model of NeP in rats [97]. On the other hand, 10 mg of oral memantine exhibited full neurobehavioral protection against the progression of neuropathy caused by cisplatin [98]. Additionally, preadministration of memantine intrathecally effectively prevented the onset of allodynia and decreased the overactivation of microglia in the spinal dorsal horn induced by SNI [99].

Clinical studies support the results of animal research, as all four studies analyzed in this review demonstrate the benefits of treating NeP with memantine, administered alone or in combination (Table 3). In a retrospective study conducted by Ahmad-Sabry et al. [100], the impact of memantine on 56 patients with CRPS (complex regional pain syndrome) was examined. The study revealed that 13 individuals achieved complete recovery, reporting a pain score of zero on the VAS scale and the absence of allodynia for at least 9 months. Additionally, 18 patients demonstrated significant improvement in reducing their VAS scores and managing symptoms of allodynia. Moreover, a randomized, blinded clinical trial indicates that administering memantine before surgery may help prevent the development of NeP 3 months post-mastectomy, as evidenced by NRS scores, impairment of cognition, and quality of life. Additionally, it hints at the potential for memantine to alleviate dysesthesia and paresthesia caused by chemotherapy [101]. Two studies examined the use of a combination of memantine and gabapentin in different types of NeP. In one study, 16 patients with PHN were treated with memantine 5–10 mg and gabapentin 300 mg, leading to a reduction in the intensity of pain [102]. Another study involved 143 patients with DN who initially received 5 mg of memantine for 1 week, followed by a combination of 10 mg memantine and 300 mg gabapentin for 8 weeks, resulting in a decrease in the severity of pain and a lowered number of patients with DN at the end of the study [103]. Both studies used the DN4 (Douleur Neuropathique 4) questionnaire to assess NeP symptoms.

### 3.4. Amantadine

Amantadine differs from other channel-blocking molecules by causing NMDAR channels to close faster [104]. Amantadine prompts NMDARs to adopt closed conformations after blocking open channels, increasing its affinity despite quick unbinding from open receptors. Therefore, amantadine primarily acts as a gating antagonist rather than a channel blocker, accelerating channel closure to stabilize closed states [104,105]. Furthermore, amantadine seems to induce the release of dopamine from brain cell nerve endings and inhibits M2 protein, which is found in viral membranes [106,107]. Amantadine is effectively absorbed through oral administration in the gastrointestinal tract; around 67% of it binds to plasma proteins, and its primary mode of excretion is through its unchanged form in the urine [108]. The most common side effects include confusion, hallucinations, tremors, seizures, nausea, and dizziness [104,109,110,111].

Although there is limited literature on the use of amantadine for NeP relief, three preclinical studies and only one clinical trial that were reviewed demonstrated the clinical benefits of amantadine (Table 5). In a rat model of NeP, amantadine reduced the hypersensitivity threshold and the frequency of hypersensitivity responses in a dose-dependent manner. Additionally, amantadine treatment decreased LP (lipid peroxidation) levels while increasing GSH (glutathione) levels in the injured tissue. The study’s data indicated that the pain-relieving effects of amantadine treatment are influenced by the reduction of oxidative stress and excitotoxicity linked to the activation of NMDAR [112]. Furthermore, Dogan et al. showed that amantadine decreased TNF-α expression in inflammatory cells surrounding the blood vessels in the substantia grisea and alba, as well as MDA and MPO (myeloperoxidase) levels. Additionally, they observed negative Bax (Bcl-2-associated X protein) expression in neuron and glial cells and positive VEGF (vascular endothelial growth factor) expression in the vascular endothelium following amantadine treatment. These findings suggest that amantadine could improve SCI by promoting angiogenesis, influencing inflammation and apoptosis, reducing oxidative stress, and modulating signaling pathways [113]. Recently, Drummond et al. [114] explored the therapeutic potential of amantadine in a rat model of CIPN. The experimental groups received oral amantadine at doses of 2, 5, 12, 25, and 50 mg/kg daily for 14 days. Amantadine significantly reduced mechanical hyperalgesia in rats treated with 25 and 50 mg/kg, indicating a dose-dependent effect. Additionally, it demonstrated anti-inflammatory properties by activating anti-inflammatory cytokines and decreasing the expression of pro-inflammatory cytokines. Moreover, amantadine exhibited antioxidant effects by enhancing the expression of the antioxidant enzymes SOD (superoxide dismutase) and CAT (catalase) and by modulating apoptotic mediators.

Azimov et al. [115] conducted a clinical trial examining the impact of amantadine, levodopa, and an amantadine–levodopa combination on 64 patients with facial nerve neuropathy. The findings indicated that both drugs had a comparable effect in reducing nerve dysfunction based on the House–Brackmann scale, with the combination showing an even greater effect.

### 3.5. Valproic Acid

Valproic acid’s mechanisms of action are multifaceted, involving inhibition of excitatory responses triggered by NMDA both in vivo and in vitro, as well as NMDA-induced convulsions in vivo, and various other facets of brain glutamatergic activity [116,117,118,119,120,121,122]. Moreover, one study demonstrated that valproic acid decreases upregulated NMDA signaling involving arachidonic acid and its metabolites in the brain [123]. Valproic acid also affects the extracellular signal-regulated kinase (ERK) pathway, non-competitively inhibits myo-inositol-1-phosphate synthetase, directly inhibits histone deacetylase (HDAC), increases GABA synthesis, and reduces GABA degradation [124,125]. Both i.v. and oral forms of valproic acid are anticipated to have similar levels of exposure, peak concentration, and minimum concentration at steady state. The drug is primarily metabolized into glucuronide conjugates, with about 30–50% being eliminated through hepatic metabolism [126]. Dose-related side effects of valproic acid include weight gain, hair loss, nausea, vomiting, and rare idiosyncratic reactions such as hematological toxicity, hepatotoxicity, pancreatitis, and polycystic ovary syndrome [127,128]. In addition to these side effects, valproic acid is a known teratogen in humans, meaning it can cause birth defects. It is associated with an increased risk of spina bifida aperta, as well as heart deformities, cleft palate, and limb anomalies [128].

All preclinical studies reviewed suggest the potential advantages of using valproic acid in treating NeP (Table 6). Out of six studies, five showed that the NMDAR antagonist was able to decrease thermal sensitivity [129,130] and mechanical sensitivity [129,130,131,132] in various animal models of NeP. Four studies indicated that valproic acid could decrease the release of cytokines like TNF-α, IL-1β, and IL-6 [129,130,132,133]. Furthermore, Chen et al. [129] highlighted that the administration of 300 mg/kg of valproic acid demonstrated an anti-neuroinflammatory effect by reducing pNFκB (phosphorylated nuclear factor-κB)/iNOS (inducible nitric oxide synthase)/COX-2 (cyclooxygenase-2) activation and preventing pAKT (phosphorylated protein kinase B)/pGSK-3β (phosphorylated glycogen synthase kinase-3β)-mediated neuronal death resulting from peripheral nerve injury in rats with CCI (chronic constriction injury). On the other hand, another animal study illustrated that valproic acid shows promise as an anti-inflammatory agent for NeP therapy by regulating microglial activity and inhibiting spinal neuroinflammation through the STAT1 (signal transducer and activator of transcription 1)/NF-κB and JAK2 (Janus kinase 2)/STAT3 (signal transducer and activator of transcription 3) signaling pathways [132]. Furthermore, Wang et al. [133] showed that chitosan nanoparticles labeled with valproic acid facilitated tissue recovery and improved locomotor function. They also enhanced neural stem cell proliferation and the expression of neurotrophic factors (BDNF (brain-derived neurotrophic factor), NGF, and NTF-3 (neurotrophin-3)), while reducing the number of microglia. Additionally, there was an increase in Tuj 1 (class III beta-tubulin)-positive cells in the spinal cord of rats with SCI, indicating that valproic acid labeled chitosan nanoparticles could potentially enhance the differentiation of neural stem cells post-SCI.

The findings from clinical studies are limited, with only one study assessing the impact of valproic acid on NeP (Table 5). In a double-blind, randomized, placebo-controlled study involving 80 patients with radiculopathy, the NMDAR antagonist was assessed in combination with celecoxib and acetaminophen. The study findings indicated that low doses of Na^+^ valproate (200 mg), particularly when combined with NSAIDs (nonsteroidal anti-inflammatory drugs), showed significant therapeutic effects in reducing or even eliminating chronic radicular pain. Pain levels were quantitatively assessed using VAS before the intervention and after 10 days [134].

**Table 6:** Preclinical and clinical studies that evaluated the effect of valproic acid on NeP.

Valproic Acid					
Preclinical Studies					
First Author/Reference	Animals	Animal Model	Dosage	Route/Frequency	Results
Chen et al. (2018) [129]	Male Sprague–Dawley rats	CCI	300 mg/kg bw	i.p. 14 days	Significantly reduced thermal sensitivity and mechanical sensitivity in plantar analgesia-meter and von Frey test; decreased pNFκB, iNOS, COX-2, pro-apoptotic protein, TNF-α, and IL-1β levels.
Elsherbiny et al. (2019) [130]	Male Swiss albino mice	Alloxan-induced DN	25–50 mg/kg bw	orally 5 days	Significantly alleviated thermal and mechanical sensitivity in hot-plate and von Frey test; decreased spinal histone deacetylases, TNF-α, and IL-1β levels.
Chu et al. (2020) [131]	Male Sprague–Dawley rats	SNI	200 mg/kg bw or 10, 20, 50 μg, in 0.5 μl	i.p. or into ventrolateral orbital cortex	Both i.p. injection and local administration demonstrated a significant analgesic effect in a dose-dependent manner in the paw withdrawal threshold test.
Wang et al. (2020) [133]	Male Sprague–Dawley rats	SCI	80 mg/kg bw	i.v. 5 days	Greatly enhanced functional recovery and tissue repair; effectively suppressed reactive astrocytes post-SCI; decreased IL-1β, IL-6, and TNF-α levels.
Wang et al. (2021) [135]	Male Sprague–Dawley rats	SCI	80 mg/kg bw	i.v.	Facilitated the recovery of tissue and locomotor function in Basso–Beattie–Bresnahan test; decreased the number of microglia; increased neural stem cell growth, BDNF, NGF NTF-3, and Tuj-1 positive cells.
Guo et al. (2021) [132]	Male Sprague–Dawley rats	SNL	300 mg/kg bw	i.p. 3 days	I.p. administration effectively reduced mechanical allodynia in von Frey test; decreased TNF-α, IL-1β, and IL-6 levels, spinal cell apoptosis, NF-κB, JAK2, STAT3; increased STAT1.
**Clinical Studies**					
**First Author/Reference**	**Population**	**Type of NeP**	**Dosage**	**Route/Frequency**	**Results**
Ghasemian et al. (2020) [134]	N = 80	Radiculopathy	Na^+^ valproate 200 mg + Celecoxib 100 mg + Acetaminophen 500 mg	orally 10 days	A low dosage of Na^+^ valproate, particularly when combined with NSAIDs, showed promising effectiveness in reducing pain, according to VAS score.

BDNF—brain-derived neurotrophic factor; bw—body weight; CCI—chronic constriction injury; COX-2—cyclooxygenase-2; DN—diabetic neuropathy; i.p.—intraperitoneally; i.v.—intravenous; IL-1β—interleukin 1β; IL-6—interleukin 6; iNOS—inducible nitric oxide synthase; JAK2—Janus kinase 2; NGF—nerve growth factor; NSAIDs—nonsteroidal anti-inflammatory drugs; NF-κB—nuclear factor-κB; NTF-3—neurotrophin-3; SCI—spinal cord injury; SNI—spared nerve injury; SNL—spinal nerve ligation; STAT1—signal transducer and activator of transcription 1; STAT3—signal transducer and activator of transcription 3; TNF-α—tumor necrosis factor α; VAS—Visual Analog Score.

### 3.6. Carbamazepine

Carbamazepine inhibits NMDA-induced Ca^2+^ influx in cultured neurons and prevents NMDA-initiated convulsions in animals [136,137]. Furthermore, the drug protects against NMDA-mediated neurotoxicity and blocks NMDA-activated membrane currents in cultured spinal cord neurons [138,139]. Additionally, carbamazepine binds to voltage-dependent Na^+^ channels, blocking action potentials that typically stimulate nerves, enhances dopamine turnover, and boosts GABA transmission [140,141,142,143]. Carbamazepine has a bioavailability of 75–85% when ingested. It undergoes significant metabolism in the liver, primarily by the CYP3A4 hepatic enzyme, which converts it to its active metabolite, carbamazepine-10,11-epoxid [144]. Carbamazepine is primarily excreted in urine as hydroxylated and conjugated metabolites, with minimal amounts of the unchanged drug [144,145]. Common side effects of carbamazepine include dizziness, ataxia, drowsiness, nausea, rash, and somnolence [146]. Agranulocytosis affects around six patients per 1 million annually, while aplastic anemia affects two patients per 1 million [146,147].

In this narrative review we investigated five preclinical studies (Table 7), with all of them demonstrating the potential of carbamazepine to reverse thermal sensitivity [148,149,150,151] and mechanical sensitivity [148,150,152], when administered alone [148,150,151,152] or in combination [149]. One study examined the impact of carbamazepine (20–40 mg/kg) given alone or with gabapentin (30–180 mg/kg) on NeP rats induced by STZ. The findings revealed that carbamazepine at 20 and 40 mg/kg did not show significant effects, but a combination of gabapentin at 90 mg/kg and carbamazepine at 20 mg/kg resulted in a notable increase in latency during the hot-plate test [149]. Dai et al. [150] investigated the effectiveness of incorporating carbamazepine into biodegradable microparticles for sustained perineural release as an analgesic for peripheral injuries. Animals treated with carbamazepine-loaded microparticles showed a two-fold increase in hindpaw withdrawal thresholds compared to controls for up to 14 days. This formulation significantly reduced systemic exposure to carbamazepine, offering substantial pain relief. In another study, the antiallodynic effects of s.c. carbamazepine, baclofen, morphine, and clomipramine were compared in an animal model of IoN-CCI (infraorbital nerve chronic constriction injury). The findings revealed that all drugs exhibited notable antiallodynic effects, with carbamazepine demonstrating the most potent effect [152].

Upon analyzing clinical studies, it was noted that, in recent years, carbamazepine has been assessed for a particular type of NeP (Table 7). Seven out of eight studies evaluated the drug’s effectiveness in treating TN, resulting in quite positive outcomes. It is not surprising, as carbamazepine has been widely acknowledged as an effective treatment for this condition for several decades, with studies dating back to 1966 [153,154]. However, two studies compared the effectiveness of carbamazepine and oxcarbazepine in TN patients indicated that, while both drugs relieved pain, oxcarbazepine demonstrated a more significant impact [155,156]. Two additional studies showed that gabapentin was more effective than carbamazepine in TN patients [157,158]. Another study indicated that the combination of carbamazepine with baclofen was more efficient and effective in pain relief, with the carbamazepine–capsaicin combination also showing better results compared to carbamazepine alone, based on the VAS scores of 45 TN patients [159].

The only study that did not assess the effectiveness of carbamazepine on TN was conducted by Khan et al. [160]. In this clinical study, the use of 200 mg of carbamazepine for 8 weeks showed positive clinical outcomes, reducing pain by approximately 80% according to VAS in 50 patients with PHN, demonstrating a potency similar to amitriptyline.

**Table 7:** Preclinical and clinical studies that evaluated the effect of carbamazepine on NeP.

Carbamazepine					
Preclinical Studies					
First Author/Reference	Animals	Animal Model	Dosage	Route/Frequency	Results
Kohli et al. (2016) [148]	Sprague–Dawley rats	CCI	20 mg/kg bw	i.p. 14 days	Reversed thermal and mechanical hyperalgesia in hot-plate and pinprick tests.
AL-Mahmood et al. (2016) [149]	Female Sprague–Dawley rats	STZ- induced DN	Carbamazepine 20–40 mg/kg bw or Gabapentin 30–180 mg/kg bw or Carbamazepine 20–40 mg/kg bw + Gabapentin 30–180 mg/kg bw	orally 1 week	Carbamazepine at doses of 20 and 40 mg/kg did not result in a notable effect on hot-plate latency. Conversely, a combination of gabapentin at 90 mg/kg and carbamazepine at 20 mg/kg led to a significant increase in latency.
Deseure et al. (2017) [152]	Male Sprague–Dawley rats	IoN-CCI	Carbamazepine 30 mg or Baclofen 1.06 mg or Morphine 5 mg or Clomipramine 4.18 mg	s.c. 1 week	All medications exhibited significant antiallodynic effects; carbamazepine demonstrated the most potent effects in directed face grooming and von Frey testing.
Dai et al. (2018) [150]	Female Sprague–Dawley rats	CCI	Carbamazepine 100 µg/mL or Carbamazepine-loaded microparticles 10–20 mg in 150 µL saline	14 days local sustained perineural release	The administration of carbamazepine-loaded microparticles resulted in more notable pain relief in von Frey and thermal plantar tests.
Bektas et al. (2019) [151]	Male Sprague –Dawley rats	Capsaicin-induced hyperalgesia	30 mg/kg bw	orally 45 min prior to capsaicin	There was a significant increase in thermal thresholds in plantar test.
**Clinical Studies**					
**First Author/Reference**	**Population**	**Type of NeP**	**Dosage**	**Route/Frequency**	**Results**
Shafiq et al. (2015) [155]	N = 202	TN	Carbamazepine 200 mg or Oxcarbazepine 200 mg	orally 8 months	Both medications alleviated pain as per VAS, with oxcarbazepine showing a more noticeable effect.
Syed et al. (2016) [161]	N = 9	TN	100–600 mg	orally 11 years	The pain perception significantly decreased according to FPS and NRS.
Puri et al. (2018) [159]	N = 45	TN	Carbamazepine 600–800 mg or Carbamazepine 600 mg + Baclofen 10–20 mg or Carbamazepine 600 mg orally + Capsaicin 0.25% cream	Carbamazepine/baclofen orally Capsaicin local application 1 month	The combination of carbamazepine with baclofen proves to be more efficient and effective in alleviating pain in patients with TN, with the carbamazepine-capsaicin combination following closely behind in comparison to carbamazepine alone according to VAS.
Kaur et al. (2018) [157]	N = 37	TN	Carbamazepine 400–1200 mg or Gabapentin 600–1800 mg	orally 3 months	Both medications demonstrated effectiveness in reducing pain, with gabapentin showing greater efficiency based on the frequency of the attacks.
Agarwal et al. (2020) [158]	N = 46	TN	Carbamazepine 400–1200 mg or Gabapentin 600–1800 mg	orally 3 months	Both drugs alleviated pain after 3 months of treatment according to VAS, with a more pronounced effect for gabapentin.
Tariq et al. (2021) [162]	N = 30	TN	100 mg	orally 28 days	The average VAS score decreased from 4.53 on day 7 to 3.27 on day 28 after treatment.
Iqbal et al. (2023) [156]	N = 56	TN	Carbamazepine 200 mg or Oxcarbazepine 200 mg	orally up to 7 months	Both medications demonstrated effectiveness based on the frequency of attacks, with oxcarbazepine showing a more pronounced effect.
Khan et al. (2023) [160]	N = 50	PHN	Carbamazepine 200 mg or Amitriptyline 25 mg	orally 8 weeks	Both drugs showed similar effectiveness, with carbamazepine reducing pain by 80% and amitriptyline by 86%, according to VAS.

bw—body weight; CCI—chronic constriction injury; DN—diabetic neuropathy; FPS—faces pain scale; i.p.—intraperitoneally; IoN-CCI—infraorbital nerve chronic constriction injury; NRS—numerical pain rating scale; PHN—postherpetic neuralgia; s.c.—subcutaneous; STZ—streptozotocin; TN—trigeminal neuralgia; VAS—Visual Analog Score.

### 3.7. Phenytoin

Studies have shown that phenytoin can effectively block NMDA responses, especially those induced by multiple applications of NMDA, as observed in research involving mouse neurons in culture [163]. Furthermore, phenytoin inhibits cortical NMDA-evoked [3H] norepinephrine efflux and NMDA-stimulated acetylcholine release from the striatum [164,165]. It has been shown that phenytoin does not affect NMDA-dependent LTP (long-term potentiation) or primed burst-induced LTP, both of which are NMDA receptor-mediated [166,167]. These findings suggest that the effects of phenytoin may be linked to its impact on voltage-dependent ion channels, while its influence on NMDA-mediated activity could be indirect or secondary. Phenytoin is fully absorbed, and approximately 90% is bound to proteins [168]. It undergoes extensive metabolism, initially converting into a reactive arene oxide intermediate. This reactive intermediate is believed to be accountable for numerous unwanted adverse effects of phenytoin [169]. The majority of phenytoin is eliminated as inactive metabolites through bile excretion [170]. Common adverse effects of phenytoin include rash, blood dyscrasias, hepatitis, nystagmus, ataxia, confusion, and memory loss [171].

This review analyzed two preclinical studies that assessed the effects of phenytoin in the CCI animal model of neuropathic pain (Table 8). Both studies showed that phenytoin effectively reversed thermal and mechanical sensitivity in rats [172,173]. Furthermore, Kocot-Kępska et al. [172] found that phenytoin reduced microglia/macrophage activation and/or infiltration at the spinal cord and dorsal root ganglion levels 7 days post-nerve injury. Significantly, the combination of phenytoin and morphine produced more effective antinociception compared to administering either drug individually.

In the clinical trials, all 12 studies demonstrated phenytoin’s effectiveness in reducing NeP (Table 8). In two studies, i.v. phenytoin was administered to patients with TN, resulting in positive outcomes [174,175]. The remaining 10 studies utilized phenytoin cream in concentrations ranging from 5–10%, with the 10% concentration yielding superior results. These studies assessed phenytoin in various types of NeP, from DN [176,177] to small fiber neuropathy [178,179,180], symmetrical painful neuropathy [181], CIAP, and CIPN [182,183]. Most studies predominantly utilized the NRS as the pain screening tool.

**Table 8:** Preclinical and clinical studies that evaluated the effect of phenytoin on NeP.

Phenytoin					
Preclinical Studies					
First Author/Reference	Animals	Animal Model	Dosage	Route/Frequency	Results
Hesari et al. (2016) [173]	Male Wistar rats	CCI	50 mg/kg bw	i.p. 14 days	Significantly reversed thermal and mechanical sensitivity in von Frey, pinprick, acetone, and hot-plate tests.
Kocot-Kępska et al. (2023) [172]	Male Wistar rats	CCI	10–60 mg/kg bw	i.p. single dose day 7 after CCI	Administered in single and repeated doses, reduced thermal and mechanical sensitivity in von Frey and cold-plate tests; effectively decreased the activation and/or infiltration of microglia/macrophages in both the spinal cord and dorsal root ganglia; the phenytoin–morphine combination resulted in superior pain relief compared to administering each drug separately.
			30 mg/kg bw	i.p. 16 h and 1 h before CCI	
			Phenytoin 30 mg/kg bw Day 8 after CCI FOLLOWED BY Morphine 10 mg/kg bw	i.p.	
**Clinical Studies**					
**First Author/Reference**	**Population**	**Type of NeP**	**Dosage**	**Route/Frequency**	**Results**
Kopsky et al. (2017) [182]	N = 1	60 years old male with peripheral NeP	5–10% cream	2 times daily 3 months	The 5% cream quickly reduced allodynia on the NRS. With the 10% cream, the person experienced complete relief from allodynia for the entire night.
	N = 1	71 years old with CIAP+CINP	5% cream	3 times daily 2 months	After the application, the patient scored 0 on the NRS.
	N = 1	54 years old with CIPN	5–10% cream	2–3 times daily 1 months	Both concentrations of the cream resulted in a reduction of pain levels on the NRS, with the 10% cream showing a more pronounced effect.
Kopsky et al. (2018) [184]	N = 70	Different types of NeP	5–10% cream	Up to 41 weeks	Resulted in a significant reduction in NeP, with more pronounced effects for the 10% concentration, according to the NRS.
Kopsky et al. (2018) [185]	N = 21	Localized NeP	10% cream	-	After 30 min, the average decrease in pain as recorded by the NRS within the region treated was 3.3.
Hesselink et al. (2017) [178]	N = 5	SFN	10% cream	-	In every instance, the time it took for the pain relief to become noticeable was less than 20 min, with four out of five cases experiencing relief within just 10 min.
Kopsky et al. (2020) [181]	N = 12	Symmetrical painful polyneuropathy	10–20% cream	6 weeks	Half of the patients exhibited positive responses to treatment on the NRS.
Hesselink et al. (2017) [179]	N = 1	SFN	10% cream	several weeks	The application of 10% cream resulted in a significant (50%) reduction in pain. The pain-relieving effects of 10% cream typically begin to take effect within approximately 5 min of application, providing relief for up to 20 h in this particular instance. The pain screening tool used was the NRS.
Hesselink et al. (2018) [183]	N = 1	CIAP	10% cream	-	Within 20 min of applying the cream, the pain in the right foot stayed constant, but the pain in the left foot decreased from a score of 7 to 2 on the NRS.
Hesselink et al. (2024) [180]	N = 3	SFN	5% cream	-	The pain experienced by two patients was significantly reduced (by more than 50%), while one patient reported complete disappearance of the pain. The pain screening tool used was the NRS.
Hesselink et al. (2016) [176]	N = 1	DN	5% cream	-	The outcome led to a significant decrease (of 50%) in neuropathic pain, according to the DN4.
Hesselink et al. (2018) [177]	N = 1	DN	10–30% cream	-	Phenytoin cream, applied in a single-blind manner, decreased pain levels on the NRS within just 5 min of application.
Schnell et al. (2020) [174]	N = 39	TN	10–20 mg/kg	i.v.	Nearly 90% of individuals experienced instant relief from pain in TN crisis.
Vargas et al. (2015) [175]	N = 1	TN	15 mg/kg	i.v.	After the infusion, the patient reported that his pain level as 2 out of 10; he was able to communicate clearly and effortlessly.

bw—body weight; CCI—chronic constriction injury; CIAP—chronic idiopathic axonal polyneuropathy; CIPN—chemotherapy-induced polyneuropathy; DN4—Douleur Neuropathique 4 Questionnaire; DN—diabetic neuropathy; i.p.—intraperitoneally; NRS—numeric rating scale; SFN—small fiber neuropathy; TN—trigeminal neuralgia.

### 3.8. Riluzole

Riluzole stabilizes voltage-dependent Na^+^ channels in their inactivated state and activates a G-protein-dependent process, leading to reduced glutamate release and inhibition of postsynaptic events mediated by NMDARs [186]. These synergistic mechanisms block excitotoxicity, providing powerful neuroprotection with minimal side effects compared to excitatory amino acid receptor antagonists. By directly and non-competitively inhibiting NMDAR activity, riluzole reduces the excitotoxic effects of excessive glutamate, thereby preventing neuronal damage and death [187,188,189,190]. Riluzole has an estimated oral bioavailability of 60%. After absorption, it is metabolized in the liver by cytochrome P450 into N-hydroxyl riluzole, which is then glucuronidated. The drug’s metabolites are mainly excreted through the kidneys, with less than 1% of the absorbed dose eliminated in urine, and about 10% of the metabolized riluzole is excreted in the feces [191]. The most frequently observed adverse effects include weakness, nausea, dizziness, cough, and abdominal discomfort [192].

Out of the 13 preclinical studies reviewed (Table 9), 7 showed that riluzole effectively reduced thermal and mechanical sensitivity in various types of NeP [193,194,195,196,197,198,199]. The only study that found riluzole to have no effect on mechanical sensitivity in an animal model of NeP in rats was conducted by Thompson et al. [200], using doses ranging from 2 to 8 mg/kg. Additionally, two animal studies highlighted that riluzole-treated rats exhibited improved motor function recovery [201,202]. The mechanisms by which riluzole provides neuroprotection are complex. They include preventing the downregulation of GLT-1 (glutamate transporter-1), the increase of glutamate concentration, and the activation of NMDAR [195], downregulating P2X7R (P2X purinoceptor 7) expression, inhibiting microglial activation [194], activating the SK (small-conductance Ca^2+^-activated K^+^) channel in the amygdala [200], inhibiting TRPM8 (transient receptor potential melastatin 8) overexpression in the dorsal root ganglions [197], and activating the GSK-3β (glycogen synthase kinase-3 beta)/CRMP-2 signaling pathway (collapsin response mediator protein-2) [203]. Furthermore, riluzole demonstrated an anti-inflammatory effect by reducing the levels of proinflammatory cytokines in rats with SCI [202]. Martins et al. [201] demonstrated that, in rats with SCI, the combination of riluzole and dantrolene provided enhanced neuroprotection by more effectively reducing apoptotic cell death compared to when each drug was administered individually. On the other hand, Ghayour et al. found that both acute and chronic administration of riluzole slowed the regeneration process and delayed the recovery of motor function rats with SNI [204].

Although animal studies suggested some advantages of riluzole in NeP, none of the three clinical trials analyzed yielded positive outcomes for riluzole therapy (Table 9). Consequently, riluzole treatment did not lead to notable improvement in patients with NeP linked to secondary progressive multiple sclerosis and cervical spine injury [205,206]. Moreover, in patients experiencing oxaliplatin-induced neuropathy, riluzole exacerbated neuropathic symptoms [207].

**Table 9:** Preclinical and clinical studies that evaluated the effect of riluzole on NeP.

Riluzole					
Preclinical Studies					
First Author/Reference	Animals	Animal Model	Dosage	Route/Frequency	Results
Karadimas et al. (2015) [193]	Female Sprague–Dawley rats	CSM	8 mg/kg bw	i.p. 2 weeks	Attenuated pain sensitivity in von Frey and tail flick tests.
Jiang et al. (2016) [194]	Male Sprague–Dawley rats	CCI	4 mg/kg bw	i.p. 5 days	Reduced thermal hyperalgesia and mechanical allodynia in plantar analgesia meter and von Frey tests; decreased the expression of P2X7R; suppressed microglial activation in the spinal cord dorsal horn.
Ghayour et al. 2017 [204]	Male Wistar rats	SNI	6–8 mg/kg bw and 4–6 mg/kg bw	i.p. single dose and i.p. 8 weeks	Acute and chronic treatment slowed the regeneration process and delayed the recovery of motor function.
Yamamoto et al. (2017) [195]	Male Sprague–Dawley rats	Oxaliplatin-induced neuropathy	12 mg/kg bw	orally 27 days	Alleviated mechanical allodynia in the von Frey test, suppressed the rise in glutamate concentration, and prevented the reduction of GLT-1 expression.
Thompson et al. (2018) [200]	Male Sprague–Dawley rats	SNL	2–8 mg/kg bw	i.p. 14 days	Inhibited vocalizations and depression-like behaviors in FST; did not affect withdrawal thresholds in the von Frey test; enhanced the mAHP mediated by SK channels in amygdala neurons.
Poupon et al. (2018) [196]	57Bl/6JRj mice	Oxaliplatin-induced neuropathy	60 μg/mL	in drinking water 28 days	Prevented cold and mechanical hypersensitivities in various tests (tail immersion, acetone von Frey, and tail brush), dexterity impairment (beam walk and adhesive removal tests), and depression-like symptoms chemotherapy (FST test); significantly prevented the decrease of NCV.
Yamamoto et al. (2018) [197]	Male Sprague–Dawley rats	Oxaliplatin-induced neuropathy	12 mg/kg bw	orally 4 days	Reduced cold allodynia in acetone test via inhibition of TRPM8 overexpression in the dorsal root ganglions.
Martins et al. (2018) [201]	Male Wistar rats	SCI	Riluzole 4 mg/kg bw or Dantrolene 10 mg/kg bw or Riluzole 4 mg/kg bw + Dantrolene 10 mg/kg bw	i.p. 15 min and 1 h before SCI	The combination synergistically enhanced neuroprotection by reducing apoptotic cell death; significantly improved motor recovery as measured by the BBB locomotor rating scale.
Zhang et al. (2018) [198]	Male Sprague–Dawley rats	SNL	12 mg/kg bw	i.p. single dose at 5 days post SNL surgery	Decreased mechanical sensitivity in von Frey test for at least 14 days; prompted LTD of spinal nociceptive signaling by acting on postsynaptic GluR2 receptors.
Wu et al. (2020) [202]	Female Wistar rats	SCI	4 mg/kg bw	i.p. 7 days	Significant increased locomotor scores (BBB score, inclined plane test); reduced spinal cavity size, increased levels of MPB and neurofilament 200; decreased levels of proinflammatory cytokines (IL-13, IL-1β, IL-6, TNF-α, TGF-β1); induced polarization of M2 microglia/macrophages.
Taiji et al. (2021) [199]	Male Sprague–Dawley rats	SNI	4 mg/kg bw	i.p. single dose at 7 days after surgery	Reduced mechanical allodynia in von Frey test.
Wu et al. (2022) [208]	Female Wistar rats	SCI	6 mg/kg bw	i.p. single dose	Decreased IL-1β mRNA, protected neurons from damage, and reduced the activation of microglia/macrophage M1 expression; increased the levels of IL-33 and its receptor ST2 in microglia/macrophages in the spinal cord.
Xu et al. (2022) [203]	Female Wistar rats	SCI	4 mg/kg bw	i.p. 7 days	Promotes neurological functional restoration by activating the GSK-3β/CRMP-2 signaling pathway.
**Clinical Studies**					
**First Author/Reference**	**Population**	**Type of NeP**	**Dosage**	**Route/Frequency**	**Results**
Trinh et al. 2021 [207]	N = 52	Oxaliplatin-induced neuropathy	50 mg	orally prior to the second oxaliplatin dose, continuing to the end of treatment	According to TNS and FACT-GOG NTX scores, riluzole worsens neuropathy symptoms, neurotoxicity, and quality of life associated with oxaliplatin treatment.
Foley et al. (2022) [205]	N = 445	NeP associated with secondary progressive multiple sclerosis	50 mg	orally 1/day for 4 weeks, then 2/day until week 96	Riluzole showed no positive effect on any NeP outcome measure (NPS and Brief Pain Inventory).
Kumarasam et al. (2022) [206]	N = 52	Cervical spine injury	100 mg FOLLOWED BY 50 mg	orally, 3 days FOLLOWED BY orally 13 days	Riluzole therapy did not result in a significant improvement in the severity of NeP, as measured by the NRS.

BBB—Basso–Beattie–Bresnahan; bw—body weight; CCI—chronic constriction injury; CRMP-2—collapsin response mediator protein-2; CSM—cervical spondylotic myelopathy; FACT-GOG NTX—Functional Assessment of Cancer Therapy/Gynecologic Oncology Group–Neurotoxicity; FST—forced swim test; GLT-1—glutamate transporter 1; GluR2—glutamate receptor 2; GSK-3β—glycogen synthase kinase-3 beta; i.p.—intraperitoneally; IL-13—interleukin 13; IL-1β—interleukin 1β; IL-33—interleukin 33; IL-6—interleukin 6; LTD—long-term depression; mAHP—medium after hyperpolarization; NCV—nerve conduction velocity; NPS—neuropathic pain score; NRS—numerical rating scale for neuropathic pain; P2X7R—P2X purinoceptor 7; SCI—spinal cord injury; SK—small-conductance Ca^2+^-activated K+; SNI—spared nerve injury; SNL—spinal nerve ligation; TGF-β1—transforming growth factor beta 1; TNF-α—tumor necrosis factor α; TNS—total neuropathy score-reduced; TRPM8—transient receptor potential melastatin 8.

### 3.9. Levorphanol

Levorphanol exhibits unique pharmacological properties by acting as a mu, delta, kappa1, and kappa3 receptor agonist, while also inhibiting the reuptake of norepinephrine and serotonin [209]. It was also found to selectively block NMDAR-mediated neurotoxicity in cortical neurons in mice and inhibit the excitatory response of rat spinal neurons to NMDA [210,211]. Its affinity for the NMDAR is stronger than methadone and comparable to ketamine. Due to its potent NMDA antagonism and specific inhibitory action on norepinephrine uptake, levorphanol is considered a strong candidate for treating NeP [212,213,214]. Levorphanol is well absorbed when taken orally and is metabolized to the inactive levorphanol-3-glucuronide. The glucuronide metabolite is excreted through the kidney [209]. Side effects include nausea, vomiting, mood and mental changes, itching, flushing, urinary difficulties, constipation, and biliary spasm [215,216].

In this narrative review, only one study, comprising two case reports (Table 10), was analyzed, and it showed promising results [217]. Pain intensity was assessed in both cases using the Edmonton Symptom Assessment System (ESAS) pain score. The first case involved a patient with osteosarcoma who was later diagnosed with phantom limb pain. Despite receiving a combination of medications consisting of hydromorphone extended-release 16 mg once daily, hydromorphone 4 mg six times a day, and gabapentin 300 mg three times a day, the pain level persists at a notable intensity, with a score of 7 out of 10 on the ESAS pain scale. However, after adding levorphanol 2 mg to the existing regimen of hydromorphone, the pain intensity decreased to 0–1 on the ESAS pain scale. The second case involved a breast cancer patient diagnosed with Brown–Sequard syndrome. Despite being on a regimen of 1200 mg of gabapentin three times daily, venlafaxine extended-release 75 mg once daily, and hydrocodone/acetaminophen 10/325 mg every 6 h, the patient continued to report uncontrolled pain. A low dose of levorphanol at 1 mg every 8 h was added alongside the existing hydrocodone/acetaminophen. After 1 month, the patient noted significant improvement in pain, reducing to a 2 out of 10 on the ESAS pain scale. This improvement continued over several months, with decreased reliance on hydrocodone until its discontinuation.

### 3.10. Methadone

Methadone is a synthetic opioid analgesic that functions as a full agonist of the µ-opioid receptor [209]. Notably, methadone also antagonizes the NMDAR and strongly inhibits the uptake of serotonin and norepinephrine, which likely enhances its pain-relief properties [209,218]. Methadone is a highly lipid-soluble opioid that is well absorbed from the gastrointestinal tract [218]. It binds extensively to plasma proteins and undergoes significant first-pass metabolism. Its elimination involves extensive biotransformation, followed by excretion through the kidneys and feces [219]. Side effects include constipation, sedation, nausea, vomiting, and respiratory depression [220].

Evidence supporting the benefits of methadone in treating NeP is derived from clinical studies, with all 10 studies included in this narrative review reporting positive outcomes (Table 11). Notably, seven of these studies focused on the effect of methadone on CRNP, with all demonstrating that the NMDAR antagonist significantly reduced pain severity [221,222,223,224,225,226,227]. Additionally, case reports by Bach et al. [228] demonstrated the safe and effective use of low-dose methadone as an adjuvant treatment in frail elderly patients with various types of NeP who could not tolerate higher doses of conventional opioids and adjuvant pain medications. Methadone helped reduce the required dosage of hydromorphone in these patients. In contrast, Madden et al. [223] demonstrated that, in children with CRNP, adding a very low dose of methadone (0.03–0.04 mg/kg) to their existing gabapentin treatment regimen effectively managed NeP syndrome. Another study compared the effectiveness of oral methadone to fentanyl patches in patients with CRNP and found that the reduction in NRS scores was significantly greater with methadone than with fentanyl [221]. Moreover, Adumala et al. [227] reported that methadone provided superior analgesic effects and good overall tolerability compared to morphine for managing CRNP. The pain reduction was measured using the NRS and DN4 questionnaires. Combining duloxetine (40–60 mg) with methadone (15–30 mg) effectively reduced CRNP and alleviated emotional symptoms in patients more than either medication used alone, as shown by ESAS scores [224].

## 4. Materials and Methods

A literature survey was conducted using PubMed to explore the most relevant articles containing preclinical and clinical research findings on the impact of various NMDAR antagonists on NeP. We restricted the search to articles that were published in English from 2015 to 2024. We used the following keywords and MeSH terms: “ketamine” OR “memantine” OR “methadone” OR “amantadine” OR “carbamazepine” OR “valproic acid” OR “phenytoin” OR “dextromethorphan” OR “riluzole” OR “levorphanol” AND “neuropathic pain” OR “neuropathy”. After careful analysis and cross-checking, we chose the most suitable studies.

## 5. Summary

Ketamine, the most extensively studied NMDAR antagonist for NeP, effectively reduced the pain sensitivity associated with NeP and exhibited anti-inflammatory potential by reducing pro-inflammatory cytokines in preclinical studies [49,50]. However, its effects may not be long-lasting [47]. Clinical studies on ketamine for NeP revealed mixed results across different administration methods. Out of 11 studies, 8 supported ketamine’s benefits. Among these, six studies used i.v. administration, with varying outcomes: three reported effective pain relief [51,52,53], one exhibited partial success [54], and two showed no significant reduction in pain [55,56]. Orally administered ketamine showed effectiveness in two studies [57,58], but a large study on CIPN yielded poor results. Topical and subcutaneous administration both showed positive outcomes in one study [60,61].

In preclinical studies, dextromethorphan effectively reversed tactile allodynia and thermal hyperalgesia [84,85]. However, combining dextromethorphan with gabapentin or oxycodone enhanced its antiallodynic effect [86,87]. Clinical studies are limited, with one study showing dextromethorphan’s antihyperalgesic effects in a freeze-injury-induced hyperalgesia model [88].

Memantine showed efficacy in preclinical studies by reversing mechanical sensitivity and reducing proinflammatory cytokine levels [96,99]. Clinical trials support its benefits in treating NeP, particularly CRPS and post-mastectomy NeP, when administered alone [100,101]. Amantadine reduced hypersensitivity and oxidative stress in preclinical studies [112,113], with positive outcomes reported in clinical trials for facial nerve neuropathy [115].

Valproic acid demonstrates potential in preclinical studies by reducing sensitivity and cytokine release [129,130]. Limited clinical evidence suggests its effectiveness when combined with NSAIDs for chronic radicular pain [134]. Carbamazepine shows efficacy in preclinical studies for thermal and mechanical sensitivity reversal, particularly in TN [148,152]. Clinical studies mainly focus on TN, with varying effectiveness compared to other medications like oxcarbazepine or gabapentin [155,157].

In preclinical studies, phenytoin effectively reversed thermal and mechanical sensitivity in rats and reduced microglia/macrophage activation post-nerve injury [172]. Combining phenytoin with morphine provided more effective pain relief than either drug alone [172]. Clinical trials demonstrated phenytoin’s effectiveness in reducing NeP, with i.v. administration benefiting TN patients [174,175] and topical phenytoin cream (5–10%) showing superior results in various types of NeP, including DN [176], small fiber neuropathy [180], and CIPN [182]. Riluzole, despite promising preclinical results [193,194,195], showed no positive outcomes in clinical trials for NeP treatment [205,207].

Levorphanol, as seen in case reports, holds promise in pain management for conditions like osteosarcoma and Brown–Sequard syndrome [217]. Clinical studies indicated that methadone is highly effective in treating NeP, particularly in cancer-related cases [224,227]. It was safe and effective at low doses for frail elderly patients, helping to reduce hydromorphone usage [228], and effectively managed pain in children with CRNP when combined with gabapentin [223]. Methadone also showed superior pain relief compared to fentanyl patches and morphine [221,227], and combining it with duloxetine further alleviated CRNP and emotional symptoms [224].

This narrative review acknowledges several limitations that warrant discussion. While preclinical studies offer promising insights into the mechanisms and potential benefits of the various NMDAR antagonists, their direct translation to clinical applications is hindered by various factors. Preclinical trials frequently use animal models that may not accurately represent human physiology and pathology. Moreover, variations in dosages, administration methods, and experimental designs across studies lead to a lack of standardized protocols.

Moreover, supplementary limitations exist within the current body of research. Many studies included in the review have small sample sizes, posing challenges in drawing definitive conclusions applicable to broader patient populations. Furthermore, there is significant variability in dosing among clinicians, leading to inconsistent treatment outcomes. Additionally, neuropathic pain encompasses a wide spectrum of conditions, meaning a pharmacologic agent that is effective for one condition may not be suitable for another. Clinicians need to recognize this variability when devising treatment plans for patients with specific types of neuropathic pain. Lastly, the considerable variation in follow-up periods among analyzed studies, ranging from weeks to months, may impact the assessment of treatment efficacy and requires careful consideration by clinicians.

## 6. Conclusions

After our thorough examination of the 10 NMDAR antagonists, it is evident that clinicians have multiple options for treating NeP. While some agents have stronger evidence supporting their efficacy, it is essential for physicians to recognize alternative choices in cases where pharmacologic drugs fail to provide sufficient relief or are limited by side effects. However, additional research is necessary to enhance understanding of the mechanisms and clinical utility of these 10 agents.

## Figures and Tables

**Table 1:** NMDAR antagonists included in this review and their molecular formulas.

Drug	Molecular Formula
Amantadine	C_10_H_17_N
Carbamazepine	C_15_H_12_N_2_O
Dextromethorphan	C_18_H_25_NO
Ketamine	C_13_H_16_ClNO
Levorphanol	C_17_H_23_NO
Memantine	C_12_H_21_N
Methadone	C_21_H_27_NO
Phenytoin	C_15_H_12_N_2_O_2_
Riluzole	C_8_H_10_FN_3_OS
Valproic acid	C_8_H_16_O_2_

**Table 3:** Preclinical and clinical studies that evaluated the effect of dextromethorphan on NeP.

Dextromethorphan					
Preclinical Studies					
First Author/Reference	Animals	Animal Model	Dosage	Route/Frequency	Results
Yang et al. (2015) [86]	Male C57BL/6J mice	SNL	Dextromethorphan 10, 20 mg/kg bw, or Oxycodone 1, 3, 5 mg/kg bw, or Dextromethorphan 10 mg/kg bw + Oxycodone 1, 3 mg/kg bw,	Dextromethorphan i.p. 14 days Oxycodone s.c. 14 days	Dextromethorphan alone did not demonstrate any notable long-term impacts. Administered in combination with oxycodone, dextromethorphan enhanced its antiallodynic effect in von Frey test.
Shi et al. (2018) [87]	Male and female Sprague–Dawley rats	Photochemically-induced ischemic SCI and SNI	Dextromethorphan 5–20 mg/kg bw or Gabapentin 7.5–30 mg/kg bw or Dextromethorphan 5–10 mg/kg bw + Gabapentin 7.5–30 mg/kg bw	i.p.	Dextromethorphan alone did not produce any pain relief in von Frey test and ethyl chloride cold test. In comparison, the dextromethorphan–gabapentin combination resulted in complete relief of allodynia, even in lower doses.
Zbârcea et al. (2018) [84]	Male Wistar rats	Vincristine-induced NeP	20 mg/kg bw	orally 7 days	Reversed tactile allodynia in Dynamic Plantar Aesthesiometer test.
Fahmi et al. (2021) [85]	Male mice	PSNL	10 nmol	intrathecally from day 8 to 14 after PSNL	Alleviated thermal hyperalgesia in stainless-steel heating plate test.
**Clinical Studies**					
**First Author/Reference**	**Population**	**Type of NeP**	**Dosage**	**Route/Frequency**	**Results**
Martin et al. (2019) [88]	N = 20	Freeze-injury-induced hyperalgesia model in healthy volunteers	30 mg	orally initially, at 5 h, 10 h, 14 h and once on day 1 after inducing the pain model	Demonstrated antihyperalgesic effects in humans, reversing sensitization in both peripheral and central neurons.

bw—body weight; i.p.—intraperitoneally; PSNL—partial sciatic nerve ligation; s.c.—subcutaneous; SCI—spinal cord injury; SNI—spared nerve injury; SNL—spinal nerve ligation.

**Table 4:** Preclinical and clinical studies that evaluated the effect of memantine on NeP.

Memantine					
Preclinical Studies					
First Author/Reference	Animals	Animal Model	Dosage	Route/Frequency	Results
Solmaz et al. (2015) [97]	Male Sprague–Dawley rats	CIP	15–30 mg/kg bw	i.p. single administration	Significantly reduced TNF-α and MDA levels and CMAP distal latency.
Ciotu et al. (2016) [95]	Male Wistar rats	Paclitaxel-induced NeP	10–30 mg/kg bw	orally 24 days	The sensitivity thresholds returned to normal levels in Dynamic Plantar Aesthesiometer test.
Chen et al. (2019) [99]	Male C57BL/6J mice	SNI	10–30 nmol	intrathecally before SNI	Preadministration of the higher dose successfully blocked the development of allodynia in von Frey and paint brush test; 10 nmol of memantine exhibited a notable impact on reducing the excessive activation of microglia in the spinal dorsal horn caused by SNI.
Salih et al. (2020) [98]	Male BALB/c mice	Cisplatin-induced NeP	5–10 mg/kg bw	orally 30 days	The higher dose showed greater efficacy in protecting against neuropathy, demonstrating full neurobehavioral protection according to open field activity, negative geotaxis, hole-board, and swimming tests.
Alomar et al. (2021) [96]	Male Swiss albino mice	Alloxan-induced DN	10 mg/kg bw	orally 5 weeks	Reduced pain symptoms in hot-plate and von Frey tests by inhibiting excessive activation of NMDAR1 receptors, lowering glutamate levels, and decreasing the release of TNF-α and IL-1β in the spinal cord.
**Clinical Studies**					
**First Author/Reference**	**Population**	**Type of NeP**	**Dosage**	**Route/Frequency**	**Results**
Ahmad-Sabry et al. (2015) [100]	N = 56	CRPS	5–10 mg increased every 4–7 days to a max. of 40–60 g	orally	A total of 13 individuals experienced full recovery, reporting a pain score of 0 on the VAS and the absence of allodynia for a minimum of 9 months; 18 patients displayed significant progress in reducing their VAS scores and managing allodynia symptoms.
Morel et al. (2016) [101]	N = 40	Mastectomy-associated NeP and CIPN	5–20 mg	orally 4 weeks, starting 2 weeks before mastectomy	At 3 months, patients exhibited a notable decrease in post-mastectomy pain intensity, as shown by the NRS. Moreover, in the group that received memantine, the symptoms of CIPN were greatly reduced.
Shaseb et al. (2023) [102]	N = 16	PHN	Memantine 5–10 mg + Gabapentin 300 mg	orally 8 weeks	The combination resulted in a decrease in the intensity of PHN symptoms, according to the DN4 questionnaire.
Jafarzadeh et al. (2023) [103]	N = 143	DN	Memantine 5 mg 1 week Followed by Memantine 10 mg + Gabapentin 300 mg or Gabapentin 300 mg 8 weeks	orally	The average DN4 questionnaire score in the memantine group was significantly lower, and the number of patients with DN in this group notably decreased by the end of the study.

bw—body weight; CIP—clinical illness polyneuropathy; CIPN—chemotherapy-induced polyneuropathy; CMAP—compound muscle action potentials; CRPS—complex regional pain syndrome; DN—diabetic neuropathy; DN4—Douleur Neuropathique 4 Questionnaire; i.p.—intraperitoneally; IL-1β—interleukin-1 beta; MDA—malondialdehyde; NMDAR1—N-methyl-D-aspartate type 1 receptor; NRS—numeric rating scale; PHN—post-herpetic neuralgia; SNI—spared nerve injury; TNF-α—tumor necrosis factor-α; VAS—Visual Analog Scale.

**Table 5:** Preclinical and clinical studies that evaluated the effect of amantadine on NeP.

Amantadine					
Preclinical Studies					
First Author/Reference	Animals	Animal Model	Dosage	Route/Frequency	Results
Dogan et al. (2019) [113]	Male Sprague–Dawley rats	SCI	45 mg/kg bw	i.p. 7 days	Decreased MDA, MPO, and TNF-α levels; neuron and glial cell showed negative Bax expression, while vascular endothelium showed positive VEGF expression after treatment.
Mata-Bermudez et al. (2021) [112]	Female Wistar rats	SCI	6.25–50 mg/kg bw	i.p. 15 days	Effectively alleviated pain-related behavior in von Frey test; decreased LP and increased GSH levels in the damaged tissue.
Drummond et al. (2024) [114]	Male Wistar rats	CIPN	2, 5, 12, 25, and 50 mg/kg bw	orally 14 days	Higher doses efficiently reduced mechanical hyperalgesia in digital analgesia-meter test in a dose-dependent manner; decreased IL-6, TNF-α, MIP-1α, Perk, Bax, Casp 3, Casp 9, and CX3CR1 expression; increased Bcl-xl, CAT, SOD, and IL-10 expression.
**Clinical Studies**					
**First Author/Reference**	**Population**	**Type of NeP**	**Dosage**	**Route/Frequency**	**Results**
Azimov et al. (2016) [115]	N = 64	Patients with neuropathy of the facial nerve	Amantadine 200 mg or Levodopa 125 mg or Amantadine 200 mg + Levodopa 125 mg	orally	There was a substantial increase in the enhancement of neurostatus dynamics when treated with the combination than with monotherapy, according to the scale of House–Brackmann.

Bax—Bcl-2-associated X protein; bw—body weight; GSH—glutathione; i.p.—intraperitoneally; LP—peroxidation levels; MDA—malondialdehyde; MPO—myeloperoxidase; SCI—spinal cord injury; TNF-α—tumor necrosis factor α; VEGF—vascular endothelial growth factor.

**Table 10:** Clinical studies that evaluated the effect of levorphanol on NeP.

Levorphanol					
Clinical Studies					
First Author/Reference	Population	Type of NeP	Dosage	Route/Frequency	Results
Reddy et al. (2018) [217]	N = 1	Phantom limb pain	Levorphanol 2 mg + Hydromorphone 4 mg every	Levorphanol every 8 h Hydromorphone every 4 h as necessary for breakthrough pain several months	One week later, pain had nearly disappeared, with a pain intensity rating of 0–1 out of 10 on the ESAS pain scale.
	N = 1	Brown–Sequard syndrome	Levorphanol 1 mg + Hydrocodone 10 mg and Acetaminophen 325 mg	Levorphanol every 8 h Hydrocodone and Acetaminophen taken as needed several months	After 1 month, pain significantly improved, scoring 2 out of 10 on the ESAS pain scale.

ESAS—Edmonton Symptom Assessment System.

**Table 11:** Clinical studies that evaluated the effect of methadone on NeP.

Methadone					
Clinical Studies					
First Author/Reference	Population	Type of NeP	Dosage	Route/Frequency	Results
Rasmussen et al. (2015) [229]	N = 1	Vincristine-induced neuropathy	32.7 mg/kg bw	i.v. 182 days	Decreased pain by as much as 4 points on the NRS scale.
	N = 1		24 mg/kg bw	i.v. 180 days	Decreased pain by as much as 5 points on the NRS scale.
Haumann et al. (2016) [221]	N = 52	CRNP	Methadone 2 mg or Fentanyl 12 µg/h	Methadone orally Fentanyl patch 5 weeks	The decrease in NRS scores was notably superior when methadone was utilized in comparison to fentanyl.
Sugiyama et al. (2016) [222]	N = 28	Severe CRNP	7.5–150 mg	orally 14 days	In this study involving patients who switched from other strong opioids like oxycodone and fentanyl to methadone, 22 patients experienced a significant reduction in their mean FPS score.
Bach et al. (2016) [228]	N = 1	A 94-year-old patient with intractable back pain secondary to spinal stenosis and disc protrusion	0.5 mg	orally every 12 h	The co-administration of methadone relieved chronic nonmalignant NeP and reduced the dosage of hydromorphone in elderly patients.
		An 88-year-old patient with phantom limb pain in right leg and NeP in the left	1–2 mg	orally every 12 h	
		A 94-year-old patient with end-stage renal disease and a C5 injury experiencing burning pain that extends from the neck down to both arms	0.5–2.5 mg	orally	
Madden et al. (2017) [223]	N = 2	Refractory CRNP in children	Methadone 0.03–0.04 mg/kg bw + Gabapentin 45 mg/kg	orally 1 year	Refractory NeP syndrome effectively managed by adding very low dose of methadone to their existing gabapentin treatment regimen.
Lynch et al. (2019) [230]	N = 9	Moderate to severe chronic NeP	5–60 mg	orally 11 weeks	All individuals demonstrated a decrease in average pain intensity based on the NPRS.
Curry et al. (2021) [224]	N = 43	CRNP	Methadone 33.75 mg (dose range) or Duloxetine 60 mg (dose range) FOLLOWED BY Methadone 15–30 mg + Duloxetine 40–60 mg	orally 2–8 weeks	After patients transitioned from monotherapy to combination therapy, there was a reduction in both the total ESAS scores and subscores. Additionally, 28% of patients on combination therapy reported a minimum two-point decrease in pain scores.
Matsuda et al. (2022) [225]	N = 3	NeP due to NBP	15–60 mg	orally 5–57 days	Pain scores decreased according to NRS in all three cases.
Fawoubo et al. (2023) [226]	N = 48	CRNP	21–60 mg	orally 28 days	By day 28, the pain intensity was notably reduced, with 53% of patients reporting a lower VAS score. Additionally, the NPSI score decreased in 50% of patients.
Adumala et al. (2023) [227]	N = 74	CRNP	Methadone 2.5–20 mg or Morphine 30–360 mg	orally 12 weeks	All participants exhibited a decrease in the average values for NRS and DN4, with a superior analgesic effect for methadone compared to morphine.

bw—body weight; CRNP—cancer-related neuropathic pain; DN4—Douleur Neuropathique 4; ESAS—Edmonton Symptom Assessment System; FPS—faces pain scale; NBP—neoplastic brachial plexopathy; NPRS—Numeric Rating Scale for Pain Intensity; NPSI—Neuropathic Pain Symptom Inventory; NRS—numerical rating scores.

## Data Availability

All data generated or analyzed during this study are included in this published article.
